# Expanding disciplinary and interdisciplinary core idea maps by students to promote perceived self-efficacy in learning science

**DOI:** 10.1186/s40594-022-00374-8

**Published:** 2022-09-10

**Authors:** Helen Semilarski, Regina Soobard, Jack Holbrook, Miia Rannikmäe

**Affiliations:** Center for Science Education, Faculty of Science and Technology, University of Tartu, Vanemuise 46, 51003 Tartu, Estonia

**Keywords:** Disciplinary core idea maps, Interdisciplinary core idea maps, Knowledge construction, Meaningful learning, Perceived self-efficacy

## Abstract

**Background:**

The goal of this research was to determine students’ perceived self-efficacy in science classes through involving students in expanding disciplinary core idea (DCI) and interdisciplinary core idea (ICI) maps, as a method to visualize knowledge (utilizing mind mapping and concept mapping) to support students to integrate interdisciplinary learning. The research involved (a) creating (by science educators) eight curriculum-related, disciplinary core idea maps and two interdisciplinary core idea maps; (b) teachers guiding students in an experimental group, to make interdisciplinary connections so as to expand DCI and ICI maps in an intervention lasting a year and a half from grade 10 to 11; (c) providing feedback on students’ developed DCI and ICI maps; (d) administering questionnaires seeking students’ perceptions about their self-efficacy towards core ideas, both before and after the intervention and (e) interviewing science teachers (5) and selected students (25), after the intervention, about their perceptions towards the use and outcomes of their DCI and ICI maps. Besides the experimental group, a control group (no intervention) was involved.

**Results:**

Outcomes showed that the intervention (guiding students in creating disciplinary and interdisciplinary core idea maps to visualize their learning) supported students significantly in their perceived self-efficacy in the fields of Life Science and Earth Science, plus in the use of Models and Systems. In Physics and Chemistry, the students’ perceived self-efficacy was not statistically significantly positive after the conducted intervention. This stemmed from disciplinary core ideas, related to Physics and Chemistry, being more abstract, with students making fewer connections and integrating less new knowledge into the related DCI and ICI maps. In the interviews, both teachers and students stated that the intervention (including expansion of DCI and ICI maps) supported students’ science learning.

**Conclusions:**

Creating and expanding disciplinary and interdisciplinary core ideas more clearly indicates students’ learning, through their ability to make meaningful connections, enabling students to raise their self-efficacy in preparing for their future. The results from this research demonstrate that students’ perceived self-efficacy can occur through knowledge visualization by expanding both DCI and ICI maps enabling the making of greater interdisciplinary connections.

## Introduction

In today’s world, scientifically literate people are needed to solve problems and make responsible decisions in science, medicine, politics, and other areas essential for society development (Organization for Economic Co-operation and Development, [OECD, 2019]). This suggests that learning in science needs to equip students not only with the necessary knowledge, but also to promote the application of knowledge, plus the gaining of twenty-first century skills and associated values (OECD, 2019), where twenty-first century skills refer to the knowledge and skills that are critically important for individuals to succeed in today’s world and in the future (Van Laar et al., 2017). These key components enhance scientific literacy (Čipková et al., 2019, 2020; Kober, 2015) and are seen as valuable in enabling science, technology, engineering, and mathematics (STEM) education (Vincent-Lancrin et al., 2019) to prepare students for yet unknown science careers, especially those demanding interdisciplinarity abilities between science and other disciplines (Darling-Hammond et al., 2020; Pleasants et al., 2021; Schleicher, 2020).

A worldwide concern in science education is perceiving learning as a series of disconnected knowledge acquisitions, which impacts on students’ interest in science (Harlen et al., 2015), or leads to a lack of perceived self-efficacy towards an ability to learn science (Semilarski et al., 2019b). In such a learning approach, students have difficulty in perceiving how to apply knowledge for solving real-life global challenges, as well as developing the ability to make (interdisciplinary) links between knowledge from multiple subjects (Schleicher, 2020; Scott, 2017; Stuckey et al., 2013).

For learning to be meaningful, Ausubel et al. (1968) indicate information needs to be conceptualized for it to be used to make connections with other previously known knowledge, thus aiding further learning. Meaningful learning enables learners to make substantive connections between new and prior knowledge (Novak, 2010). These connections facilitate storage of acquired knowledge in long-term memory, causing personalized and continuous learning (Ausubel, 1968; Heddy et al., 2017). As indicated in previous research, encouraging students to relate their previous knowledge to new knowledge can have a positive influence on their perceived self-efficacy and can promote students’ meaningful learning (Baltaoğlu & Güven, 2019; Zang & Soergel, 2014).

Disciplinary core ideas (DCIs) and interdisciplinary core ideas (ICIs) are central and necessary for scientists to explain phenomena and solve ill-structured problems. As such, DCIs and ICIs form a unified scientific framework for organizing the curriculum, as, for example, the topics set out in the Estonian curriculum (Semilarski et al., 2019b). Within science learning, DCIs and ICIs form a necessary structure for conceptualizing science and for supporting students’ perceived self-efficacy—including relevant interconnections between prior and new knowledge (NRC, 2012; Semilarski et al., 2019b; Soobard et al., 2018). Disciplinary core ideas and interdisciplinary core ideas are also seen as important in everyday life, both now and in the future (AAAS, 2001; Semilarski et al., 2019b). One potential approach to support students’ perceived self-efficacy is to interrelate new and existing knowledge, supporting interdisciplinary learning (Linn, 2006; Shen et al., 2016). The integration of DCIs and ICIs both within and across subjects (e.g., being multi- and inter-disciplinary), promoting knowledge integration (Linn, 2006), supports the development of wider conceptualizations (supported by making connections), which, in turn, make the learning process more society related and meaningful (NRC, 2012; Sukhov et al., 2018).

Findings from earlier studies show that conceptualization of the learning can help students give meaning to their collective experiences and thus improve their perceived self-efficacy (students’ beliefs in their capabilities [Bandura, 1986]) as well as encourage meaningful learning (Weick et al., 2005).

This research aims to identify students’ ability to use disciplinary and interdisciplinary core ideas to construct diagrammatic maps of the knowledge needed to support their perceived self-efficacy in science and thus promote meaningful learning. DCI and ICI maps are methodological tools for supporting students’ conceptualization and providing a framework for prior and new knowledge related to disciplinary or interdisciplinary core ideas.

Research questions

The following research questions are put forward:

**RQ1** How effective are students in expanding DCI and ICI maps as a tool for promoting perceived self-efficacy in science?

**RQ2** What differences occur in students’ perceived self-efficacy between an experimental group that expand DCI and ICI maps and a control group not utilizing such maps?

**RQ3** What are students’ and teachers’ perceptions of the developed teaching/learning method, within the experimental group, for supporting students’ perceived self-efficacy?

## Literature review

### Knowledge construction

Knowledge can be taken as the ability to use ideas and to be able to (know how to) use knowledge acquired through experience or education forming the theoretical or practical understanding of a subject (Cukurova et al., 2018; Ericson, 2002; Howell et al., 2014; Jonassen et al., 2003). A process in which knowledge is constructed by students through an individual’s interactions with the world and through social interactions is defined as knowledge construction (Chapman, 1999). Anderson and Krathwohl (2001) as well as Alavi & Leidner, 2001, see the goal being to integrate ideas and experiences into meaningful dimensions and principles for explaining phenomena and solving everyday problems, resulting in a deeper understanding of the subject matter.

Learning is a process of constructing knowledge utilizing twenty-first century skills, through connecting thoughts, or, possibly, through an investigation process (Houwer et al., 2013). More specifically, students learn by connecting new ideas and experiences with knowledge already obtained, thereby constructing new meaning (NRC, 2012). It is thus not surprising that within education there is a need to focus on how to facilitate students’ learning, i.e., how to make it meaningful for them, and hence enable students to make connections (Ausubel, 1968; Bransford et al., 2000; Heddy et al., 2017).

The theory of knowledge construction is based on constructivism (Wilson, 2001; White, 2001). Constructivism in teaching and learning is grounded on the assumption that teaching is student-centered (Wilson, 2001; White, 2001). Teachers recognize the value of students’ prior knowledge and support learners in making links among new ideas and experiences with the knowledge they already possess through using active and personally meaningful activities, or situations for learning, plus providing opportunities for collaboration and understanding of their level of cognition, referred to as perceived self-efficacy (Piaget, 1972; Thompson, 2000; Vygotsky, 1978). Perceived self-efficacy relates to people’s beliefs about their capabilities to produce effects and to succeed (Bandura, 1986).

### Meaningful learning

Meaningful learning is grounded on the theory of Ausubel (1963) and Novak’s (1993, 2002) theory of human constructivism. These researchers draw on the constructivist process of learning as connecting new ideas and experiences to prior knowledge so as to develop new knowledge (Freedman, 1994; Tasker, 1992; Wilson, 2001), allowing steps facilitating the learning to be comprehensive and lifelong (Ausubel, 1968; Heddy et al., 2017; Novak, 2002). Research suggests that students connect knowledge most effectively in active classrooms characterized by students participating in discourse and in learning processes (e.g., discussing ideas in group work, suggesting approaches and undertaking experimentation, arguing so as to reach a consensus resolution related to meaningful problems/issues) thus expanding their meaningful connections, as well as encouraging students to reflect and talk about their learning (Mayer et al., 1996; Thompson, 2000).

Meaningful learning and constructivism are not only important from the students’ perspective, but also rely on the teacher to support students’ learning by appropriate planning of lessons, facilitating students’ active participation, i.e., students taking a more active role in their learning (Thompson, 2000). Teachers need to be aware that students, as novice learners, often possess a less developed, or incomplete knowledge construction (Kober, 2015). Thus, teachers need to assist students’ knowledge constructions so that students become expert learners, whereby prior and new knowledge are deeply interconnected and easily retrieved (Ambrose et al., 2010).

A key factor in meaningful learning is enabling students to make connections that allow learning to be comprehensive and lasting throughout their lives (Heddy et al., 2017; Kober, 2015). Ausubel’s theory of meaningful learning focuses on three main characteristics (Ausubel et al., 1978):

1. relevant prior knowledge—to engage students to make connections between new ideas and experiences and prior knowledge.
2. meaningful material—to engage students in using materials that are interesting and relevant to their needs and,
3. the learner must choose to learn meaningfully—to engage students in opportunities to express and explore their learning experience, thoughts, and arguments.

Of these three features, it is clear that only the second is fully within the educator’s control. While the choices of educators need to support students in relating new ideas and experience to their prior knowledge, educators can only indirectly influence the third characteristic by developing materials that build sufficient interest in new knowledge, encouraging students to actively make connections to prior knowledge (Bretz et al., 2013; DeKorver & Towns, 2015; Merriam & Clark, 1993). Thus, through successive interactions, a learner’s prior knowledge can progressively develop new meaning, become richer, more refined, and capable of serving as an anchor for new meaningful learning (Ausubel, 1963). Previous research shows that of all the factors that influence learning, the most important is connecting to a student’s prior knowledge; hence a key aspect considered as the starting point for teaching (Bretz et al., 2013).

### Knowledge visualization

While meaningful learning is the constructive process of making meaning of the world, enabling the students to use knowledge in new and unfamiliar situations (Mayer, 2002; Odden & Russ, 2019), it does not emphasize the hierarchical nature of knowledge, nor does it explain how students’ conceptualizations develop and change over time (Jena, 2012).

Meaningful learning can be promoted through knowledge visualization (Weick et al., 2005). Mind maps and concept maps can be used for organizing and representing knowledge, i.e., connecting new knowledge to prior knowledge (Bressington et al., 2018). When learners visualize their knowledge using mind maps and concept maps, meaningful learning is facilitated because prior knowledge is interrelated with new ideas and experiences (Cañas & Novak, 2019; Novak, 2010). Findings from previous research have outlined that a mind map’s hierarchically structure enables students to appreciate how their knowledge develops, thus leading to meaningful learning (Bressington et al., 2018; Buzan, 2009a, 2009b; Novak, 2010). Also, students who can make connection are more successful in regular knowledge-based school tasks and assessments (Kubsch et al., 2020; Nordine et al., 2019).

For teachers, mind maps and concept maps can help identify students’ prior knowledge, enabling better reflection of their teaching approach and determining more appropriate teaching materials for supporting students’ progress (Ausubel et al., 1978; Heddy et al., 2017; Kalyuga & Sweller, 2004; Novak, 2010). For students, knowledge visualization helps to develop coherent, deeply connected, transferable, conceptual frameworks about the ideas under study (Ambrose et al., 2010; Buzan, 2009a; Novak, 2010). A more hierarchical structure for mind maps and concept maps indicates more meaningful learning. Previous research has also highlighted that knowledge visualization in the teaching process is effective, with students developing more extensively and thematically organized maps and more richly portraying interconnectedness of knowledge in their maps (Dhindsa et al., 2010; Jena, 2012; Mystakidis, 2019). Also, meaningful learning requires tasks linked to an authentic experience or real-world context so that the teaching becomes personally significant and transferable (Howell et al., 2014; Mystakidis, 2019).

### Disciplinary and interdisciplinary core ideas

Disciplinary and interdisciplinary core ideas in science teaching are fundamental for making sense of phenomena and solving complex problems (NRC, 2012; Semilarski et al., 2019b, 2020), such that:

- disciplinary core ideas are the science content that students need to know and be able to apply and which are specific to a science field, such as core ideas in Life Science (hereditary, genetic variation), Earth Science (relief formation, climate and weather), Chemistry (atoms and molecule, chemical reactions) and Physics (energy conversion, movement: waves), and
- interdisciplinary core ideas are transferrable across different science fields, e.g., models, systems, etc., yet are central for learning. These are much broader in scope and are not necessarily rooted solely in science. These support how students think like scientists, focusing on a specific aspect of the observations. These are a way of interconnecting the different science subjects.

The use of DCIs and ICIs offers an organization of knowledge which enables conceptualization, raise science-related career awareness, and prepare students for deeper scientific inquiry and further conceptualization (Duncan et al., 2016; Krajcik & Delen, 2017; NRC, 2012). DCIs and ICIs have been seen as a key perspective for organizing knowledge regarding energy conversion, or genetic variation.

DCIs and ICIs have broad importance within and across science disciplines, are relatable to globally challenging concerns and provide a key tool for conceptualizing and investigating complex situations (Harlen et al., 2015; NRC, 2012). Recognizing and conceptualizing DCIs and ICIs is especially important for students and teachers when relating knowledge from different science areas to solve problems, or explain phenomena (Harlen et al., 2015; Semilarski et al., 2020). Science content conceptualization, using DCIs and ICIs, supports students’ perceived self-efficacy and allows students to apply their knowledge in different situations to solve complex problems and make responsible decisions (Duncan et al., 2016; Krajcik & Delen, 2017).

To visualize DCIs and ICIs, it is reasonable to use knowledge visualization and a constructivist approach. Thus core idea maps can be considered as tools for promoting knowledge integration (AAAS, 2001; Semilarski et al., 2021a). Core idea maps are methodological teaching and learning tools, which depict development of conceptualization of important DCIs and ICIs in the sciences through different school levels. These DCI and ICI maps pay attention to the related knowledge, skills and the development of career awareness to support students perceived self-efficacy and meaningful learning. However, it is important to consider the students’ and teachers’ beliefs in the use of appropriate teaching methods, as these can influence students’ achievements in science, including information processing, reasoning and decision-making (Fischer & Hänze, 2020; Lee et al., 2016; Zlatkin-Trotschanskaia et al., 2020).

According to previous studies, students’ perceived self-efficacy tends to be higher towards DCIs related to Life Science and Earth Science and lower towards DCIs related to Chemistry and Physics, where the knowledge tends to be more abstract (Cheung, 2015; Jamil & Mahmud, 2019; Semilarski et al., 2019a; Soobard et al., 2018).

#### Perceived self-efficacy

The psychologist Albert Bandura has defined perceived self-efficacy as the gained perception of students’ beliefs in their capabilities to exercise control over their own functioning and over events that affect their lives (Bandura, 1986). One’s sense of perceived self-efficacy can provide the foundation for well-being and personal accomplishment (Bandura, 1986). Furthermore, progress by students in their learning is connected with their perceived self-efficacy toward their competence in interacting with DCIs (Semilarski et al., 2019b; Smit et al., 2019; Wu & Fan, 2017). Thus, to deeply engage students in science learning, perceived self-efficacy is seen to be of great importance (Lin, 2021). Students, perceived to possess higher self-efficacy, set higher goals and expend more effort towards their achievement and show a higher level of thinking about conceptualizing science (Smit et al., 2019). Students’ perceived self-efficacy is seen as the key to promoting students’ engagement and learning (Wu & Fan, 2017).

As science education today is seen as focusing on preparing future citizens who are able to think critically and not merely recipients of facts, both meaningful science learning and the students’ perceived self-efficacy to undertake such learning are perceived as essential in the learning process (Baltaoğlu & Güven, 2019; Vincent-Lancrin et al., 2019).

## Methodology

This study was carried out to identify students’ ability to expand disciplinary and interdisciplinary core idea maps to support their perceived self-efficacy across science subjects.

### Sample

The samples consisted of an (a) experimental group (209 students, and 12 teachers, undertaking the intervention from five schools) and (b) a control group, receiving no intervention (162 students also from five schools). The intervention was carried out for 18 months from January 2019 to June 2020 involving students from grade 10 and 11 (Table 1).

**Table 1:** Overview of the participants in the intervention

School	No. of students	No. of teachers	Lessons taught by teachers
School A	59	2	Biology and Chemistry
School B	25	3	Biology, Chemistry, and Physics
School C	54	2	Biology and Earth Science
School D	36	2	Chemistry and Physics
School E	35	3	Biology, Chemistry, and Earth Science

The control group was chosen according to similar characteristics (school location and number of students, teachers who participated in professional development courses) as the experimental group. Students in the control group participated in responding to a questionnaire on perceived self-efficacy towards DCIs and ICIs after the intervention was completed in the experimental schools.

### Study design

Before the intervention, one teacher from each school (a total of 5) participated in a 4-day (24 h) professional development workshop. All teachers who participated in the workshop also collaborated with other science teachers from their schools and shared their experiences and knowledge. This was seen as important for promoting science teachers’ collaboration and to bring about interdisciplinary interconnections. During the workshop, participating teachers:

- received an overview of the disciplinary and interdisciplinary core ideas (both the nature of the DCIs and ICIs and their importance).
- listened to researchers make presentations about the DCIs and ICIs in their field, e.g., a university professor talked about climate change.
- received an overview of the meaning and scope of twenty-first century skills and career awareness, regarding aspects to promote together with core ideas (Semilarski et al., 2021a)
- practiced knowledge visualization using mind mapping and concept mapping.
- practiced creating interdisciplinary DCI and ICI maps.
- collaborated with other science teachers to find interconnections between topics covered in their subjects.
- received an overview of how to integrate DCI and ICI maps into their teaching and how to actively involve students in the process of expanding disciplinary and interdisciplinary core idea maps.

As part of the professional development, the teachers received exemplary lesson plans, including everyday related scenarios, 8 completed DCI maps and 2 completed ICI maps (following the knowledge visualization process). These DCI and ICI maps had been previously developed through collaboration between science educators and teachers who did not participate in the intervention. Besides this, the teachers received a guide on how to instruct students to develop DCI and ICI maps*.* Approximately one hour of the workshop was spent providing teachers with an overview of the intervention—procedures, measures, logistics. The science teachers were shown how to guide their students to connect their prior and new scientific ideas (including making interdisciplinary connections), thus associating them with twenty-first century skills and science-related careers, with the purpose of raising students’ perceived self-efficacy.

Although the 8 DCI and 2 ICI maps received by teachers included knowledge progression across different grade levels (grade 1–12), the maps provided to the teachers for use with grade 10–11 students only included knowledge progression from grade 1–9. The knowledge progression from grade 1–9 was given to students to determine scientific ideas they were expected to have learned, although students had an opportunity, during their science lessons, to add more ideas illustrating their prior knowledge in the maps. During the intervention, teachers handed out these DCI and ICI maps to the students in grades 10–11 and instructed them to expand these for grade level 10–12 and to add more prior knowledge to the DCI and ICI maps for grades 1–9. The purpose was for students to fill in the DCI and ICI maps with their new knowledge and by relating it to their previous knowledge. The students were also given the task to expand on how the knowledge was interconnected to twenty-first century skills and to science-related careers. During the intervention, students filled in the DCI and ICI maps in their different science (biology, geography, chemistry, and physics) lessons by including more interdisciplinary connections.

**Fig. 1:**
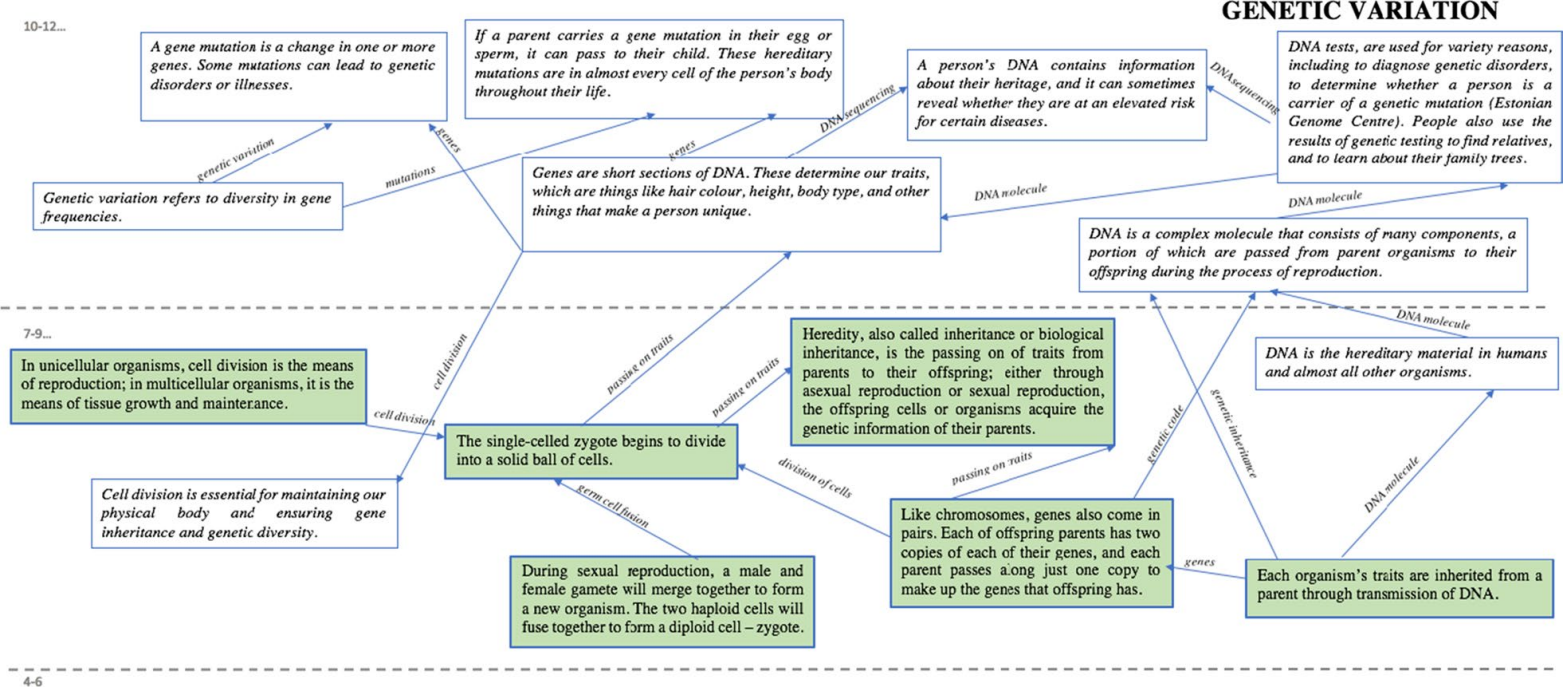
Part example of students expanded disciplinary core idea map, based on AAAS (2001)

Figure 1 provides an overview of how students expanded DCI and ICI maps in which anticipated grade level 7–9 knowledge progressions were provided (green boxes). Thus, the students did not create this part, but they included more boxes so as to include, in an appropriate manner, their previous knowledge. For the grade level 10–12 students, the DCI and ICI maps were completed by the student (white boxes). Associated with the DCI and ICI maps, students also received a list of careers, related to the corresponding core idea.

Throughout the intervention, all teachers (a total of 12) participated in mini-seminars before any new set of materials (DCI and ICI maps; a total of 10) were provided, encouraging collaboration among the teachers. During the meetings held during the intervention period, all teachers shared their experiences, suggestions and the problems encountered, with solutions being sought collaboratively.

### The intervention

To identify suitable core ideas, which students could use to expand within disciplinary core idea maps, the researchers initially chose 32 core ideas from the conceptual strand maps in the Atlas of Scientific Literacy (AAAS, 2001) seen as relevant within the Estonian national curriculum (2011). Then the 10 most important core ideas (important to everyday life and in the future) were chosen by 12 science teachers and science educators by identifying, in their opinion, the two most important core ideas for each of the four science disciplines (forming a total of 8) and the two most important interdisciplinary core ideas. A Google Form questionnaire was administrated to each teacher and educator, and they were requested to make their own selection of core ideas based on the following:

1. perceived as critical for a future workforce.
2. perceived as important across the science disciplines.
3. associated with the Estonian national curriculum, and
4. applicable across multiple grades at increasing levels of depth and sophistication.

Based on the highest frequency, the following 8 disciplinary core ideas were selected by the researchers:

1. Life Science—genetic variation (enables natural selection, one of the primary forces driving the evolution of life) and DNA/heredity (biological processes by which characteristics are transmitted from parent to their offspring).
2. Earth Science—weather/climate (states of the atmosphere) and land surface changes (waves, wind, ice, water shape and reshape the Earth’s land surface).
3. Chemistry—characteristics of substances (help to identify and classify substances) and chemical reactions (processes that lead to the chemical transformation), and.
4. Physics—energy conversion (processes of converting one type of energy into another form) and motions/waves (movement of a distortion of a material or medium).

The following two interdisciplinary core ideas were also identified: models (dimensional representation of a person, taught or structure—typically on a smaller scale than the original) and systems (an organized scheme or method). While a system is was seen as an organized group of related objects or components, models were considered and could be used for conceptualizing and predicting the behavior in systems (NRC, 2012).

The selection of the 8 disciplinary and 2 interdisciplinary core ideas chosen for this intervention, were published in previous research conducted by Semilarski et al. (2021b). These core ideas were linked to form a scientific framework for various curriculum topics set out in the Estonian curriculum (Estonian Government, 2011). During the intervention, the corresponding disciplinary and interdisciplinary core idea maps were expanded by students.

**Fig. 2:**
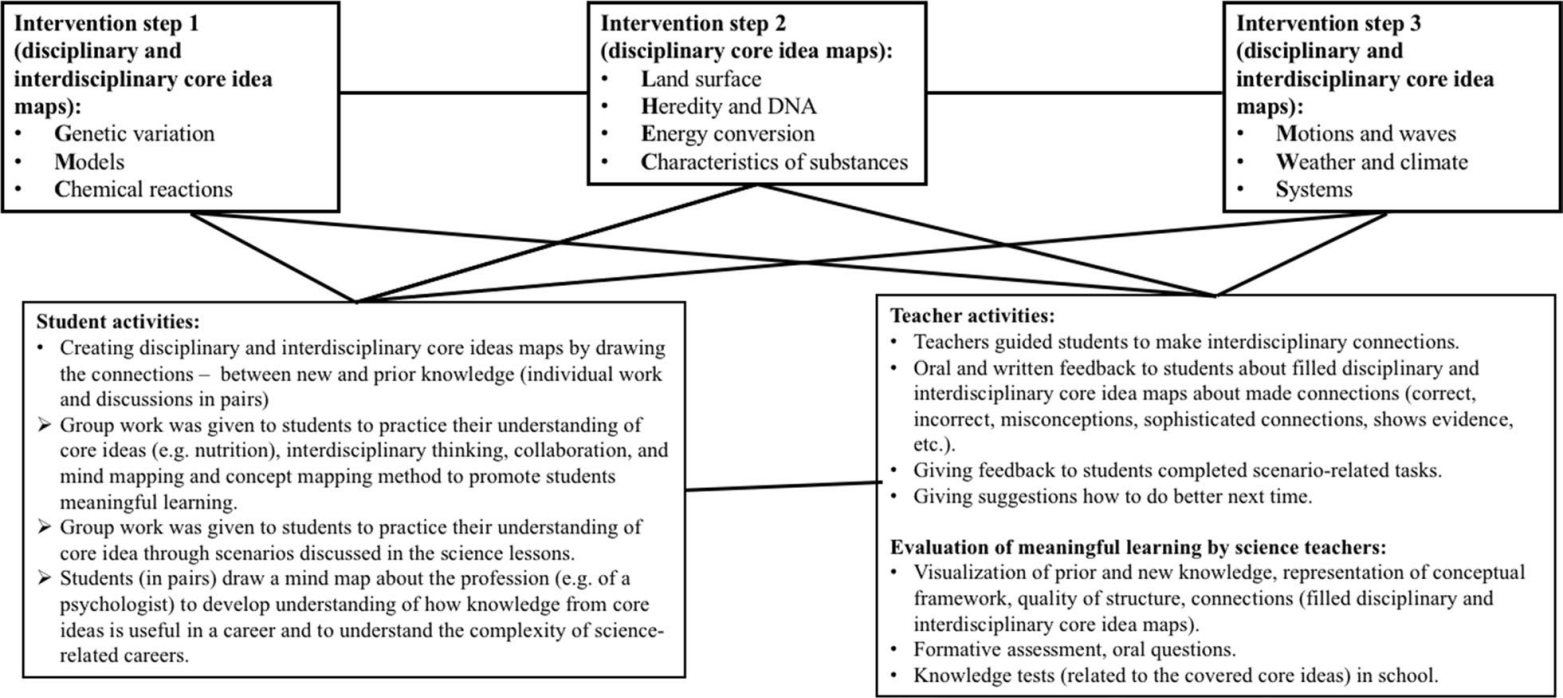
Intervention design

An overview of the intervention design (content, activities, and reflection about the method) is as presented in Fig. 2. The intervention was divided into three steps, each step concentrating on a specific disciplinary and interdisciplinary core idea map. For each disciplinary and interdisciplinary core idea map, the teaching occupied at least 6 lessons.

### Instruments development

Two types of instruments were used for data collection—(a) a questionnaire (experimental and control group) and (b) interviews (experimental group).

- Questionnaire
  A pre- and post-questionnaire (Semilarski et al., 2019a; Soobard et al., 2018) was used for determining students’ perceived self-efficacy, related to the core ideas. This was administered to obtain an overview of the effectiveness of the intervention.
  All questions were answered using a 4-point scale ranging from 1- “I do not agree at all” to 4- “I definitely agree”. While the pre-questionnaire was administered by paper and pencil, the post-questionnaire used a Google Form template. This made it possible to collect data during the COVID-19 epidemic when schools in Estonia were operating in a virtual mode.
  The two questionnaires and their parts, the number of questions, and from whom data were collected, are as shown in Table 2.
  The control group questionnaire was administered only at the end of grade 11 because previous research about students’ perceptions towards competence in core ideas was shown similarly in many studies carried out with different students (Semilarski et al., 2019b; Soobard et al., 2018). These studies conducted in different years showed that there was no change in the perceived self-efficacy of upper secondary school students.
- Interviews
  Interviews were conducted with the experimental group students and teachers (separately) to determine their perceptions of the developed method (students expanded core idea maps). The interview questions (Table 3) were developed and validated by the researchers. Two experts, who were familiar with the topic, gave critical comments on the interview guide and the interview questions were refined accordingly. Next, a pilot interview was carried out with one teacher (who did not participate in the study) through Zoom before the interviews were conducted to check time needed for the interviews and to make further refinements. This also allowed a check on the interview techniques being used (Dictaphones, Zoom recordings, etc.), and to practice interviewing skills. The key interview questions are as shown in Table 3.

**Table 2:** Overview of the questionnaires

Questionnaire	Questionnaire parts	No. of questions	Data collection
Pre-questionnaire	Part 1: students’ perceived self-efficacy towards core ideas in science	23	Experimental group and Control group
Post-questionnaire	Part 1: students’ perceived self-efficacy towards core ideas in science	23	Experimental group
	Part 2: the usefulness (including the combination of like, interest, importance, etc.) of implemented core idea maps in science lessons—10 core ideas as part of the intervention	10

**Table 3:** The key interview questions related to teachers and students’ perceptions of the implemented DCI and ICI maps for promoting students’ self-efficacy

Students (*N* = 25)	Teachers (*N* = 5)
Did you find it useful to expand DCI and ICI maps? Explain	Did you find it useful for students to expand DCI and ICI maps? Explain
Did you collaborate with your classmates when you expanded the DCI and ICI maps? Explain	Did you collaborate with your teacher colleagues, when students expanded the DCI and ICI maps? Explain
What feedback did you receive from teachers when you expanded DCI and ICI maps?	What feedback did you give to students about their expanded DCI and ICI maps? Do you have any suggestions about how to give feedback to students expanded DCI and ICI maps?
Which DCI and ICI maps were most useful for you? Explain	Which DCI and ICI maps were most useful for you as a teacher? Explain
Did you think expanding DCI and ICI maps were useful for you in your science studies? Explain	Did you think expanding DCI and ICI maps were useful for students in their science studies? Explain
	With which core ideas, did students indicated more prior and new knowledge and made more connections?

All interviews were conducted using video conferencing, and were recorded and fully transcribed. Before each interview, students or teachers were asked for their consent to be recorded.

### Data collection

Students participating in the study provided consent as required from all participating schools and their school heads. All collected data were stored, keeping in mind the principles of confidentiality. The participation by students and teachers was voluntary.

Table 4 is compiled to show the instruments used and an overview of the data collection process.

**Table 4:** Overview of the data collection per instrument used

	Instrument	Time when carried out	Approximate duration (in minutes)
Experimental group	Pre-questionnaire	January 2019	20–25
	Post-questionnaire	May 2020	20–25
	Interviews (with students)	May–June 2020	25–45
	Interviews (with teachers)	June 2020	20–45
Control group	Post-questionnaire	May 2020	15–20

### Data analysis

A mixed-method (Creswell, 2012) approach to data analysis was considered the most pertinent for this research, based on the collected data (both quantitative and qualitative).

### Questionnaire

To analyze the quantitative data, descriptive statistics (mean, standard deviation, statistical significance) and reliability were conducted using SPSS version 24 (Ghufron & Ermawati, 2018). The mean scores of students’ perceived self-efficacy towards competence in core ideas were compared and analyzed using a paired sample t-test. However, before the data were analyzed for significant difference, its normality and homogeneity were tested using the Lilliefors formula for normality and the homogeneity test was carried out using a Bartlett-type formula. The statistical program Mplus (Version7) (Muthén & Muthén, 1998–2015) was used for confirmatory factor analysis (CFA). CFA was used to raise the interpretability of the entire questionnaire and results with respect to the internal structure (Lewis, 2017).

The data from the experimental (pre and post) and control group (post) questionnaire were first tested for normality by using the Lilliefors formula (data were normally distributed and shown to fit a bell-shaped curve). Also, a homogeneity test was conducted by using the Bartlett test with all three sets of questionnaire data. Based on the result of homogeneity testing, it was shown that the value of χ_o_^2^ was lower than χ_t_^2^ for all three data sets. Because χ_o_^2^ was lower than χ_t_^2^, it was concluded that the collected data were homogeneous.

### Interviews

The qualitative data from interviews were analyzed descriptively, following the approach proposed by Patton (1990). For in-depth analysis, students’ and teachers’ answers were coded using inductive thematic analysis, taken as a standard content analysis approach (Patton, 1990). The themes identified were strongly related to the collected data themselves. In this research, coding themes were used, after transcribing the conducted interviews, to gain a more detailed perspective of what occurred based on the purpose of the research.

### Validity and reliability

The process of triangulation was conducted by comparing and analyzing the results of the interview and questionnaire simultaneously (Patton, 1990). Both questionnaire and interview questions were validated by the experts (science teachers and educators). An overview of how the validity and reliability of the various data sources were determined is as indicated in Table 5.

**Table 5:** Validation and reliability of the created instruments for this research

Instrument	Validity/reliability	Used validation/reliability method
Pre- and post-questionnaire	Content validity	Expert opinion method: an agreement by 12 independent experts in the field of science education as to whether the content of a measure covers the full domain of the content
	Construct validity	Analysis of the Estonian secondary school science curriculum and syllabus to ensure items are valid in terms of expected learning outcomes. For data analysis CFA was used
	Reliability	Cronbach alpha = 0.82 over the sample (with each factor over 0.74). CFA was used to test whether measures of the construct are consistent with a researcher’s understanding of the nature of that construct (factors)
Interviews	Content validity	Inductive thematic analysis was used to analyze the transcripts of the interviewers´ answers
	Construct validity	Themes identification and labeling
	Inter-coder reliability	The percentage agreement between two coders (science educators) was, with students’ interviews, 86% and teachers interviews, 78%. For resolving disagreements, the coders negotiated toward consensus. Coders made estimations and negotiated their response before reporting the final outcome (Epley & Gilovich, 2006)

## Results

### Students’ perceived self-efficacy towards disciplinary and interdisciplinary core ideas in the experimental group

The conducted Confirmatory Factor Analysis (CFA) revealed similar changes in each of the five areas for the disciplinary or interdisciplinary core ideas, identified as:

- Life Science
- Earth Science
- Chemistry
- Physics
- Models and Systems.

The comparison of perceived self-efficacy toward disciplinary and interdisciplinary core ideas after the intervention (Table 6) showed that the grade 11 students’ perceived their self-efficacy towards their competence related to DCIs was significantly higher than before the intervention in the areas of Life Science, and Earth Science, as well as Models and Systems. In Chemistry and Physics, the change was not statistically significant.

**Table 6:** A pre- and post-questionnaire results comparison using CFA on students’ perceived self-efficacy towards disciplinary and interdisciplinary core ideas, for the experimental group (*N* = 209)

The groups of disciplinary and interdisciplinary core ideas	Factor loadings		Pre		Post		Paired sample t-test			Sig. level 0.05
	Pre	Post	*M*	SD	*M*	SD	*t*	*df*	SE	
Life Science										
Cell functions in tissues	0.64	0.54	2.51	0.78	3.32	0.80	10.48	416	0.08	< 0.05
Aerobic and anaerobic respiration			2.69	0.68	2.99	0.88	3.89	416	0.08	< 0.05
Heredity and DNA*			2.77	0.83	3.57	0.83	9.85	416	0.08	< 0.05
Genetic variation*			2.71	0.73	3.41	0.79	9.41	416	0.07	< 0.05
*M*			***2.64***	***2.67***	***3.32***	***0.83***	***8.35***	***416***	***0.08***	** < *0.05***
Earth Science										
Rainforest deforestation	0.62	0.63	2.85	0.91	2.95	0.80	1.19	416	0.08	> 0.05
Land surface change*			2.60	0.77	3.00	0.89	4.91	416	0.08	< 0.05
Weather and climate*			2.82	0.76	3.12	0.70	4.20	416	0.07	< 0.05
Natural hazards			3.02	0.68	3.13	0.65	1.69	416	0.07	> 0.05
Climate warming			2.86	0.83	3.09	0.83	3.83	416	0.08	< 0.05
Solar and lunar eclipse			2.82	0.80	3.00	0.87	2.20	416	0.08	< 0.05
*M*			***2.83***	***0.79***	***3.05***	***0.79***	***2.85***	***416***	***0.08***	** < *0.05***
Chemistry										
Chemical reactions*	0.54	0.67	2.43	0.92	2.51	0.93	0.88	416	0.09	> 0.05
Natural phenomena at the particulate level			2.40	0.85	2.49	0.89	1.06	416	0.09	> 0.05
The nature of interactions between bodies			2.46	0.87	2.50	0.90	0.46	416	0.09	> 0.05
Characteristics of substances*			2.44	0.87	2.54	0.97	1.11	416	0.09	> 0.05
*M*			***2.43***	***0.88***	***2.51***	***0.92***	***0.91***	***416***	***0.09***	** > *0.05***
Physics										
Electricity generator	0.51	0.58	2.40	0.91	2.40	0.71	0.00	416	0.08	> 0.05
Motions and waves*			2.36	0.86	2.56	0.86	1.12	416	0.08	> 0.05
Energy conversion*			2.37	0.89	2.47	0.84	1.18	416	0.09	> 0.05
*M*			***2.38***	***0.89***	***2.48***	***0.80***	***1.21***	***416***	***0.08***	** > *0.05***
Models and Systems										
Systems*	0.71	0.70	2.37	0.86	3.25	0.86	10.46	416	0.08	< 0.05
Cause and effect			2.64	0.82	3.13	0.86	5.96	416	0.08	< 0.05
Natural and human-made systems			2.51	0.85	3.30	0.95	8.95	416	0.09	< 0.05
Structural properties of the objects			2.33	0.87	3.25	0.87	10.81	416	0.09	< 0.05
Models*			2.38	0.85	3.26	0.85	10.90	416	0.08	< 0.05
*M*			***2.45***	***0.85***	***3.24***	***0.88***	***9.33***	***416***	***0.09***	** < *0.05***

*Disciplinary and interdisciplinary core ideas used in the intervention research; significance level 0.05; M-mean; *SD* standard deviation; t-statistics; *df*-degrees of freedom; SE-standard error of the difference, measured on 4-point Likert-type scale

Table 6 shows students’ perceived self-efficacy towards the various sub-groups in each of the disciplinary and interdisciplinary core ideas (* marks the disciplinary and interdisciplinary core idea part of the intervention).

In the area of Life Science, students’ perceived self-efficacy was significantly higher towards all disciplinary core ideas at the end of the intervention in grade 11. More specifically:

- in Earth Science, students’ perceived self-efficacy was significantly higher towards two disciplinary core ideas that were part of the intervention (land surface, weather/climate) and two disciplinary core ideas that were not part of the intervention (climate warming, solar/lunar eclipse). For two other DCIs (rainforest deforestation, and natural hazards), students’ perceived self-efficacy did not change significantly.
- in Chemistry and Physics, perceptions toward competence in disciplinary core ideas did not change significantly despite the intervention.
- in Models and Systems, perceived towards interdisciplinary disciplinary core ideas was significantly higher towards all DCIs at the end of the intervention.

### Comparison between experimental and control group perceived self-efficacy towards disciplinary and interdisciplinary core ideas after the conducted intervention

The experimental and control group comparison (shown in Appendix 1: Table 9) revealed different outcomes:

- In Life Science, students' self-efficacy toward three disciplinary core ideas (cell functions, hereditary, and genetic variation) is statistically significantly higher in the experimental group than in the control group. There was no significant difference in perceived self-efficacy in one DCI (aerobic and anaerobic respiration).
- In Earth Science, students' perceived self-efficacy towards four DCIs (land surface, weather and climate, natural hazards, climate warming) is statistically significantly higher in the experimental group than in the control group. In two DCIs (rainforest deforestation, solar and lunar eclipse), there was no significant difference in perceived self-efficacy in both groups.
- In Chemistry, there was no statistically significant difference between the intervention and control group for all DCIs (chemical reactions, particulates, interactions between bodies, characteristics of substances).
- In Physics, all differences between the intervention and control group were statistically significant (electricity generator, motions, waves, and energy conversion).
- Within Models and Systems, all differences between intervention and control group were statistically significant (systems creation, causes and effects, natural and human-made systems, structural properties of the object, models).

When analyzing the change in perceived self-efficacy towards disciplinary and interdisciplinary core ideas used in the intervention, students’ perceived self-efficacy towards all disciplinary core ideas (except Chemical reactions) was higher in the experimental group compared to the control group.

### Students’ perceptions about the usefulness of students expanded disciplinary and interdisciplinary core idea maps

Table 7 shows students’ agreement (*M* ≥ 2.50) or disagreement (*M* ≤ 2.50) regarding the usefulness of expanding the DCI and ICI maps. The groups of disciplinary core ideas were created based on the factor analysis.

**Table 7:** An evaluation by the experimental group students (*N* = 209) of the usefulness of expanding DCI and ICI maps

The group of disciplinary core ideas	Implemented DCI map	*M*	SD
Life Science	Genetic variation	3.21	0.78
	Heredity and DNA	3.11	0.89
Earth Science	Land surface changes	2.98	0.80
	Weather and climate	3.78	0.85
Chemistry	Chemical reactions	2.45	0.77
	Characteristics of substances	2.56	0.85
Physics	Motions and waves	2.67	0.71
	Energy conversion	3.01	0.91
Models and Systems	Models	3.51	0.90
	Systems	3.01	0.78

*M mean, *SD *standard deviation, measured on 4-point Likert-type scale

According to this analysis, the most useful DCI maps were Weather and Climate, Models and Genetic variations. At the same time, the least useful were Chemical reactions, Characteristics of substances, and Motions and waves.

To obtain a more detailed overview of the usefulness of expanding DCI and ICI maps, in terms of improved conceptualization in school science, 25 students were interviewed after the intervention. The identified themes were supported by extracts from the conducted interviews and were presented based on students’ and teachers’ descriptions of the expanded DCI and ICI maps. Pseudonyms were used for the interviewed students and teachers, to ensure their anonymity.

In general, students indicated they found their expanding DCI and ICI maps useful. They perceived the DCI and ICI maps as novel, interesting and supportive of active participation and meaningful learning.

*“I liked all these activities. Different activities supported my learning in such a way that I could connect my previous knowledge to the new knowledge. We also had ‘joint’ lessons. It was interesting and exciting to see how teachers from different lessons worked together.” *(Thomas).

Students agreed that their expanding DCI and ICI maps forced them to collaborate with their classmates (in undertaking the expanding of DCI and ICI maps and presenting outcomes).

*“We collaborated more with our classmates than before. It was interesting to find solutions to problems and exchange ideas with others. I also felt that sharing my ideas and listening to ideas from other classmates supported my own self-efficacy and meaningful learning.” *(Anni).

Students agreed that expanding DCI and ICI maps was useful because they were able to present their understanding of science to their teachers. Students also agreed that expanding DCI and ICI maps was interesting because they developed their own DCI and ICI maps and in so doing recognized connections between concepts. They also indicated that their motivation in science lessons was higher compared with regular teacher-centered lectures.

“I liked connecting knowledge and concepts. Maps helped me to link my previous knowledge to the new knowledge.” (Sam).

*“I do not like when my teachers only talk about new topics. And I enjoy the lessons where we work mostly with the maps. I like that we learn more about careers and skills and connect these to the core idea maps.” *(August).

For students, the DCI maps seen as most useful were the Heredity and DNA maps because they believed the ideas in these maps were highly related to everyday life and were complex. Students also indicated other interesting DCI and ICI maps, such as:

- Models, because it was interesting and simple, and it was easy to understand.
- Weather and climate, because it was strongly related to the everyday life, it was an interesting topic to cover in the science lessons.
- Genetic variation, because it was interesting, understandable and a topic with which students had familiarity.

*“The heredity map was the most important to me. I feel that this was an issue that always surrounded us. For example, genetic testing, and whether to do it or not.”* (Cathy).

*“Weather and climate, as it was linked to the climate change. Nowadays, it was very real, and we also participated in a ‘climate change strike’, and this motivated us to learn more about it in our lessons.”* (Harry).

*“Certainly, heredity because it was very fascinating. Since this was a rather complicated topic, the use of this map helped make the topic clearer.” *(Jane).

In general, students agreed that expanding DCI and ICI maps supported their studies in science. They reasoned that the DCI and ICI maps supported their understanding of the scientific ideas. Students’ responses further indicated that expanding their DCI and ICI maps raised their self-confidence towards learning the topic and raised their motivation to study science.

*“Yes. For example, the map of chemical reactions helped me to better understand this topic. And I realized that it was not just about chemistry either, e.g., in the nutrition task, our group found that it was also related to chemical reactions.”* (William).

*“Yes. Now I ask more in class about heredity if I don’t understand something, or if there is something of interest to me.” *(Juliet).

*“Yes. Before I was not so motivated to study science, but these maps were interesting.” *(Alex).

Furthermore, in general, students:

- liked to collaborate with others
- welcomed the opportunity to elaborate their understanding of scientific ideas
- perceived DCI and ICI maps as interesting
- recognized the DCI and ICI maps promoted higher motivation towards science learning.

### Teachers’ perceptions about the usefulness of students’ expanded disciplinary and interdisciplinary core idea maps

During the intervention teachers monitored the process by for example looking over students´ expanded DCI and ICI maps.

The monitoring process during the intervention is as shown in Table 8.

**Table 8:** Teacher (6) responses related to monitoring the students expanded DCI and ICI maps

Question	Teacher responses
How many connections did students make in their expanded DCI and ICI maps about genetic variation?	Students made many connections (over 15) in their expanded DCI and ICI maps and connected their prior and new knowledge
How many connections did students make in their expanded DCI and ICI maps about chemical reactions?	Students made few connections (less than 8) in their expanded DCI and ICI maps and struggled to connect their prior and new knowledge
On which students expanded DCI and ICI maps did students indicate more science-related career?	More careers were outlined on students expanded DCI and ICI maps with—eather and Climate, Heredity (and DNA), and with Models and Systems
On which students expanded DCI and ICI maps did students indicate less science-related career?	Very few careers were outlined on students expanded DCI and ICI maps with—Chemical reactions and Motions: waves
Which students expanded DCI and ICI maps were interconnected with models?	With Heredity (DNA), Weather and Climate, and with Motions and Waves
Which students expanded DCI and ICI maps were interconnected with systems?	With Heredity (DNA) and with Weather and Climate

Five teachers were interviewed after the intervention. The interviews indicated that in general, teachers found DCI and ICI maps useful and that they believed the maps supported students’ learning. They also felt that during the intervention they were able to play an active, cooperative role. Teachers agreed that:

- DCI and ICI maps provided study materials.
- they were interested in DCIs and ICIs.
- that the DCI and ICI maps supported the collaboration between teachers and students.

*“It was an exciting experience. Maps made lessons interesting.” (Mike).*

*“With this new approach, I found that I had a good collaboration with my colleagues, with whom we were able to incorporate each other’s lesson content into our lessons.” *(Sandra).

All interviewed teachers agreed that they collaborated with other teachers.

*“As a Life Science teacher, I get along well with a physics teacher because we have a good relationship. This is why I cooperate with her.” *(Sandra)

*“I maintain a good connection with my school physics teacher. I share the school’s laboratory.” *(Minnie).

Teachers indicated that DCI and ICI maps supported students’ knowledge construction and helped to reveal students’ misconceptions.

*“Oh, exciting maps. Through these, my lessons become more exciting. When you give a student a colored map to start filling out, it is always interesting, because it is something we do not traditionally do in science.” *(Mike)

*“I agree that it is important to emphasize that they have learned similar topics in middle school. When I ask them about genetic variability, they are silent and have nothing to say. But when I give the Genetic variation map to them, they remember that they have learned it before and it is easier for them to discuss it with me in the lesson.” *(Jane).

One teacher pointed out that the creation of expanded DCI and ICI maps by students on Heredity and DNA, Genetic variation, and Models maps were useful, because she was a Life Science teacher, and, in her opinion, these DCI maps were strongly related to her field. On the other hand, another teacher said that the Energy conversion map was the most useful DCI map. In his opinion, everything was based on energy, and this had good potential for interdisciplinary teaching for all disciplines. Yet another teacher pointed out that Models and Systems maps were useful because students had an interest in these ICI maps and were able to demonstrate independence while expanding their maps.

*“The applied maps illustrate well why it is important to study science. Especially the heredity map, because students are interested in what makes them unique, why some diseases run in their families, etc.” (Minnie).*

*“Certainly, understanding the models is most important. Models help us to understand the world, e.g., the globe which is a model illustrating how the Earth appears and over the years, globe models have improved. Thus, it is important to emphasize to students that models can always be improved.” *(Jane).

All teachers agreed that according to their perception, DCI and ICI maps were useful for students in their science studies to support students’ perceived self-efficacy.

*“Students’ results improved, and they were more willing to cooperate with me and other classmates. During the intervention, students became increasingly confident in making connections and relating new knowledge to prior knowledge.” *(Mike).

All teachers agreed that students expanded DCI and ICI maps in more depth (added more new scientific ideas and interconnections on the expanded DCI and ICI maps) related to Models and Systems, Earth Science and Life Science. Teachers also indicated that in relation to the disciplinary core ideas in Chemistry and Physics, the students expanded DCI and ICI maps were significantly less extensive.

## Discussion

The goal of this research was to explore the effectiveness of supporting students’ perceived self-efficacy in science classes so as to enable students to be capable of meaningfully expanding basic disciplinary and interdisciplinary core idea maps, based on their visualization of knowledge gained and their ability to make interdisciplinary connections.

### Perceived self-efficacy towards disciplinary and interdisciplinary core ideas in the experimental group

Students higher perceived self-efficacy was triggered through teaching methods involving students expanding disciplinary and interdisciplinary core idea maps (knowledge visualization, interacting with scenarios, undertaking group work, etc.). More specifically, the knowledge visualization, involving the construction of knowledge (interrelating prior and new knowledge), was the most frequently reported source of triggering and sustaining meaningful learning (Duncan et al., 2016; Holley & Park, 2020).

Based on the changes in students’ perceived self-efficacy towards disciplinary core ideas and from the conducting of interviews with both teachers and students, the findings showed that these interactions were more likely to enhance students’ perceived self-efficacy when elaborating core ideas within Life Science, Earth Science, and Models and Systems. This finding was in line with previous studies (Holley & Park, 2020), which determined:

‘the more retrieval paths students create and use while learning, the deeper the new knowledge becomes, and the more confidence students are about their own learning competence’.

In the interviews, the teachers indicated that in the areas where students’ perceived self-efficacy was higher, students were more active in making interdisciplinary connections, the disciplinary and interdisciplinary core ideas were more relevant to students’ everyday life, and these were linked to science-related careers.

However, in the subjects of Chemistry and Physics, this learning approach was less supportive of meaningful learning. One possible reason for this was that in Chemistry and Physics students lacked the ability to add a diversity of interconnections in the disciplinary core idea maps. Teachers reported that significantly fewer connections (including interdisciplinary connections) were made by students. Furthermore, the related disciplinary core ideas were not seen as relevant and important. This finding was similar to studies by Bartimote-Aufflick, (2016), Krajcik and Delen (2017) and Sukhov et al. (2018), which showed that new learning is more likely to occur when students saw it as useful for their future, as well as more interesting when building interdisciplinary connections while learning, and when acquiring new knowledge.

In interviews with students, based on the formulation of disciplinary core ideas in Life Science, Earth Science and Models and Systems:

- Students stated in the interviews that these disciplinary and interdisciplinary core ideas were more interesting and important to them, it was easier to make more interconnections between their prior and new knowledge. This supported previous studies which sought to enhance students’ perceived self-efficacy towards disciplinary core ideas, but without an intervention (Bartimote-Aufflick et al., 2016; Semilarski et al., 2019; Soobard et al., 2018).
- Not only did students show significant self-efficacy gains in the overall factors, but changes were also significantly positive with most of the disciplinary and interdisciplinary core ideas developed. Not surprising, these DCIs and ICIs tended to place an emphasis on everyday life and were less abstract. However, exceptions were noted, associated with self-efficacy gains in Rainforest deforestation and Natural hazards (Earth Science, Table 3), where pre-questionnaire results had already indicated students held a high perceived self-efficacy.
- Positive attitudes supported the use of DCI and ICI maps. This was in line with previous studies indicating students’ perceived self-efficacy was higher when students were active participants in the learning process, and the focus was on understanding and using knowledge, rather than just remembering facts (Bartimote-Aufflick, 2016; Thompson, 2000; Novak, 2010). Students’ perceived self-efficacy was also supported where students were able to exhibit reasoning skills and put forward logical solutions for problems, as well as, being confident in the learning process (perceived self-efficacy) (Holley & Park, 2020).

In the intervention, the learning built on students’ prior knowledge (knowledge visualization), the development of meaningful materials (enhancing of the initial core idea maps), encouraging collaboration (group work), and encouraging interdisciplinary connections so as to support students’ perceived self-efficacy (Holley & Park, 2020). But, even so, it was still difficult to change students’ low perceptions about their self-efficacy in Chemistry and Physics.

The outcomes also indicated that it was important that students were able to recall prior learning and how to relate new scientific ideas to their prior knowledge. Such action helped students to create a comprehensive picture of knowledge development in these subjects and this, in turn supported students’ perceived self-efficacy, agreeing with Bartimote-Aufflick, (2016).

The findings were seen as important in pointing to the need to support students interdisciplinary learning in Physics and Chemistry and to seek ways on how to connect these ideas to other scientific ideas in different disciplines. Also, it was important to note that, in this research related to Chemistry and Physics teaching, the chosen disciplinary core ideas were more abstract (i.e., characteristics of substances, motion and waves, etc.) This might be one of the reasons why students’ perceived self-efficacy was low, as outlined by the teachers during the interviews. However, the findings did support previous studies where the students’ perceived self-efficacy tended to be lower towards disciplinary core ideas related to Chemistry and Physics, and where the learning tended to be more abstract (Cheung, 2015; Jamil & Mahmud, 2019; Semilarski et al., 2019b; Soobard et al., 2018). Previous research also showed that students had a lower perceived self-efficacy toward Chemistry knowledge in general (Cheung, 2015; Jamil & Mahmud, 2019; Semilarski et al., 2019b).

The use of disciplinary and interdisciplinary core idea maps was seen as unique because during the learning process, students themselves were enabled to recall their previous knowledge and relate it to new scientific ideas by expanding their disciplinary and interdisciplinary core idea maps. More important, however, was this approach confirmed that meaningful learning took place, as well as a positive increase in students’ perceived self-efficacy towards core ideas (Ausubel, 1968; Ausubel et. al., 1978). An implication, in for future research, is exploring such an approach to promote meaningful learning in all science subject areas. A key factor for achieving this could be the integration between different science disciplines and involving students in more real-world problems and phenomena they experience.

### Comparison between experimental and control group students’ perceived self-efficacy towards disciplinary and interdisciplinary core ideas

In the teaching of Life Science and Earth Science, students’ perceived self-efficacy among most disciplinary core ideas used in the intervention was significantly higher when compared with the control group. However, it was noted that no statistically significant difference was found among the development of three disciplinary core ideas (aerobic and anaerobic respiration, rainforest deforestation, solar and lunar eclipse), which although not included in the intervention study, gained high perceived self-efficacy in both groups. This could be seen as pointing to the effectiveness of the intervention and that the use of disciplinary and interdisciplinary core idea maps as a learning approach, emphasizing the integration between different science lessons, could support students’ perceived self-efficacy (Holley & Park, 2020; Novak, 2010). On the other hand, in guiding the development of core ideas on Models and Systems, the perceived self-efficacy of the experimental group students was significantly higher at the end of grade 11 than for the control group students in promoting all related single core ideas. This could be linked to the interdisciplinarity (NRC, 2012) of Models and Systems and that students´ higher perceived self-efficacy towards these, after the intervention, indicated that there was a need to more effectively integrate science learning. This was seen as in line with that advocated by other researchers (Darling-Hammond et al., 2020).

The research results indicated that the experimental group was better placed when interrelating disciplinary core ideas, but the control group was equally up to the task when conceptualizing any one single core ideas as separate entities. This outcome supported a major advantage of developing disciplinary and interdisciplinary core idea maps in that providing opportunities for students to make connections between DCIs and ICIs led to higher perceived self-efficacy related to the discipline and could promote more meaningful students’ learning (Ausubel, 1968; Ausubel et. al., 1978; Novak, 2010).

### Students’ perceptions of the developed method for supporting their perceived self-efficacy

Students who indicated they found expanding disciplinary and interdisciplinary core idea maps useful, explained that they liked it, found it novel, interesting and gave the possibility to actively participate. This was in line with previous research, which showed that students associated “like” with novel experiences in the classroom (Zlatkin-Trotschanskaia et al., 2020). Nevertheless, some students did not like the development of disciplinary and interdisciplinary core idea maps, linking their dislike to a particular lesson, or group work session. This supported previous research confirming these aspects could be associated with dislike against the method of teaching (Zlatkin-Trotschanskaia et al., 2020). This was supported by previous research, associating students’ and teachers’ beliefs with influencing students’ achievements (Fischer & Hänze, 2020; Zlatkin-Trotschanskaia et al., 2020).

Students pointed out that using disciplinary and interdisciplinary core idea maps over one and a half years raised their self-confidence towards their competence in science lessons. This was similar to other research that indicated that a constructivist science teaching approach led to positively changes in students´ science achievements (Holley & Park, 2020).

### Teachers perceptions of the developed method for supporting students’ perceived self-efficacy

Teachers indicated that they found this developed method useful because it allowed them to collaborate with other teachers and raise students’ awareness about disciplinary and interdisciplinary core ideas. Previous research had also found that teachers recognized the importance of collaboration and supporting each other (Berebitsky & Salloum, 2017; Mowafaq et al., 2019). The teachers also indicated that they appreciated the teaching–learning materials, which were provided during the intervention.

Furthermore, in general, teachers:

- found disciplinary and interdisciplinary core idea maps to be interesting and important for them in teaching and supporting students’ learning in science.
- noted that disciplinary and interdisciplinary core idea maps helped them to determine students’ misconceptions in particular topics.
- agreed that disciplinary and interdisciplinary core idea maps were useful for students in learning based on their experience and,
- wanted more disciplinary and interdisciplinary core idea maps on other teaching areas to implement in their schools.

Teachers also appreciated that the collaboration with colleagues could lead to students’ being better guided to making interdisciplinary connections between knowledge areas (Davies & Delvin, 2010), or overcoming misconceptions (Harlen et al., 2015). If science was divided between separate subject lessons, an emphasis was needed on integrating knowledge for students from each science subject and to promote insight about the world, as well as demonstrating their knowledge within and across core ideas (Scott, 2017).

## Conclusion

This research sought to provide empirical evidence how the implementation of expanding disciplinary and interdisciplinary core idea maps as a method might enhance students’ perceived self-efficacy. In general, the method in which students expanded DCI and ICI maps was seen as effective and supported students’ perceived self-efficacy in Life Science, Earth Science, and with Models and Systems. Reasoning for this was that in these areas it seemed easier for students to recall what they had learned previously. But, although positive tendencies were found within Chemistry and Physics, the change in students’ perceived self-efficacy was not statistically significant.

The comparison between the experimental and control group confirmed that the intervention had a positive change on students’ perceived self-efficacy towards disciplinary and interdisciplinary core ideas.

The outcomes from the conducted interviews revealed that, in general, students’ and teachers’ perceptions of the developed method for supporting students’ perceived self-efficacy was positive. They felt that the DCI and ICI maps helped to support students’ meaningful learning. Both teachers and students stated in their interviews that knowledge construction tasks (knowledge visualization through mind mapping and concept mapping, handling scenarios, making interdisciplinary interconnections) helped students to better link prior knowledge to new knowledge.

## Limitations

A small sample size of students and schools were used as a convenient sample and therefore results were not generalizable to the whole population. Further studies with a larger number of participants might provide more conclusive results.

Pre- and post-questionnaires were conducted using a 4-point Likert-type scale. This provided an opportunity to research students’ opinions on the positive and negative sides. However, students did not have an opportunity for indicating a neutral perspective. Not all components of disciplinary and interdisciplinary core ideas were measured in this study. This was not considered possible using a paper and pencil, large scale questionnaire. The developed questionnaire also did not include any open-ended questions, which could have the advantage of offering a wide range of responses that helped to capture students answers.

## Recommendations

In order that science education is more integrated, more attention needs to be paid to teaching approaches and how these support students’ perceived self-efficacy and meaningful learning. Based on the research outcomes, it is noted that teaching material needs to relate to establishing disciplinary and interdisciplinary core ideas and supporting students’ knowledge construction. Thus, to infuse meaning into the lessons, it is important to make the learning content as meaningful as possible.

Students need to be provided with opportunities to construct their knowledge (such as by drawing mind maps and concept maps) that are interconnected and contextualized in such a way that students are able to call upon the ideas being taught at a subsequent time.

The findings of such studies can be used to also further theorize about the developmental use of disciplinary core idea maps and provide practical recommendations for curriculum design and classroom practices which aim to enhance students’ perceived self-efficacy in science. Although the preliminary results are promising there is still a perceived need for follow-up research to further explore the efficacy of the various components of this intervention stage.

Further research is needed to study more deeply how well using students expanded disciplinary and interdisciplinary core idea maps can promote students’ meaningful learning.

## Data Availability

All data generated or analyzed during this study are included in this published article and its Additional files.

## Appendix 1

See Table 9.

**Table 9:** Experimental (*N* = 209) and control group (*N* = 162) CFA on students’ perceived self-efficacy towards DCIs and ICIs (grade 11) after the conducted intervention (May–June 2020)

The group of disciplinary and interdisciplinary core ideas	Factor loadings		Exp		Con		Paired sample *t*-test			Sig. level 0.05
			M	SD	M	SD				
	Exp	Con					*t*	*df*	SE	
Life Science										
Cell functions in human tissues	0.54	0.44	3.32	0.80	2.85	0.70	5.92	369	0.08	< 0.05
Aerobic and anaerobic respiration			2.99	0.88	2.95	0.64	0.49	369	0.08	> 0.05
Heredity and DNA*			3.57	0.83	2.84	0.66	9.17	369	0.08	< 0.05
Genetic variation*			3.41	0.79	2.83	0.63	7.65	369	0.08	< 0.05
Earth Science										
Rainforest deforestation	0.63	0.55	2.95	0.80	2.90	0.73	0.62	369	0.08	> 0.05
Land surface change*			3.00	0.89	2.50	0.52	6.36	369	0.08	< 0.05
Weather and climate*			3.12	0.70	2.30	0.66	11.47	369	0.07	< 0.05
Natural hazards			3.13	0.65	2.60	0.65	7.79	369	0.07	< 0.05
Climate warming			3.09	0.83	2.15	0.57	12.33	369	0.08	< 0.05
Solar and lunar eclipse			3.00	0.87	2.76	0.60	3.00	369	0.08	> 0.05
Chemistry										
Chemical reactions*	0.67	0.72	2.51	0.93	2.38	0.73	1.46	369	0.09	> 0.05
Natural phenomena at the particulate level			2.49	0.89	2.43	0.81	0.67	369	0.09	> 0.05
The nature of interactions between bodies			2.50	0.90	2.40	0.78	1.12	369	0.09	> 0.05
Characteristics of substances*			2.54	0.97	2.37	0.85	1.77	369	0.10	> 0.05
Physics										
Electricity generator	0.58	0.63	2.40	0.71	2.05	0.59	5.06	369	0.07	< 0.05
Motions and waves*			2.56	0.86	2.32	0.59	3.04	369	0.08	< 0.05
Energy conversion*			2.47	0.84	2.17	0.60	3.85	369	0.08	< 0.05
Models and Systems										
Systems*	0.70	0.67	3.25	0.86	2.52	0.60	9.20	369	0.08	< 0.05
Cause and effect			3.13	0.86	2.65	0.69	6.16	369	0.08	< 0.05
Natural and human-made systems			3.30	0.95	2.05	0.66	14.29	369	0.09	< 0.05
Structural properties of the objects			3.25	0.87	2.25	0.67	12.11	369	0.08	< 0.05
Models*			3.26	0.85	2.30	0.65	11.92	369	0.08	< 0.05

^*^Disciplinary core ideas used in the intervention research; *Significance level 0.05; *M*-mean; SD-standard deviation; t-statistics; *df* degrees of freedom; SE-standard error of difference; measured on 4-point Likert-type scale
